# Optimal Timing for Oocyte Denudation and Intracytoplasmic Sperm Injection

**DOI:** 10.1155/2012/403531

**Published:** 2012-02-20

**Authors:** Catherine Patrat, Aida Kaffel, Lucie Delaroche, Juliette Guibert, Pierre Jouannet, Sylvie Epelboin, Dominique De Ziegler, Jean-Philippe Wolf, Patricia Fauque

**Affiliations:** ^1^Laboratoire de Biologie de la Reproduction, AP-HP, Hôpital Bichat Université Paris Diderot, 46 Rue Henri Huchard, 75018 Paris, France; ^2^Laboratoire de Biologie de la Reproduction, AP-HP, Hôpital Cochin, 75014 Paris, France; ^3^Service de Gynécologie-Obstétrique, Institut Mutualiste Monstsouris, 75014 Paris, France; ^4^Service de Gynécologie-Obstétrique II, AP-HP, Hôpital Bichat, 75018 Paris, France; ^5^Service de Gynécologie-Obstétrique II, AP-HP, Hôpital Cochin, Université Paris Descartes, 75005 Paris, France; ^6^Laboratoire de Biologie de la reproduction CECOS, CHU de Dijon, Université de Bourgogne, Dijon, France; ^7^Institut Curie, CNRS UMR 3215, INSERM U934 Paris, France

## Abstract

*Objectives*. To analyze the impact of oocyte denudation and microinjection timings on intracytoplasmic sperm injection (ICSI) outcomes. *Study Design*. We included ICSI cycles with the following parameters: rank 1 or 2, female age <36 years, male factor infertility, long protocol using GnRH agonist and rFSH for ovarian stimulation, and use of freshly ejaculated sperm (*n* = 110). Several ICSI parameters were analyzed according to the time between oocyte retrieval and denudation (*T*_1_) and the time between denudation and ICSI (*T*_2_) using a statistical logistic regression analysis. *Results*. Neither *T*_1_ nor *T*_2_ had a significant influence on the Metaphase II (MII) rate but the fertilisation rate (FR) showed a significant improvement when *T*_1_ was longer (optimal results at *T*_1_ = 3 hours) while FR significantly decreased with the increase of *T*_2_. Optimal implantation (IR) and pregnancy (PR) rates were obtained when *T*_1_ was around 2 hours. *Conclusion*. Incubation of oocytes around 2 hours between retrieval and denudation may not increase MII rate but appears to lead to the optimal combination of FR and IR.

## 1. Introduction

Intracytoplasmic sperm injection (ICSI) is the treatment of choice for couples with severe male infertility. The microinjection technique has been completely standardized but there is no common standard for the precise timings of all the procedures. No more than 7 studies focusing on the influence of ICSI procedure timings on the outcoming results were published [1–8], with discrepancies in the conclusions. 

Although it has been shown that incubation of oocytes for 2–6 h prior to IVF (*In Vitro* Fertilization) improves fertilization and pregnancy rates [9–12], there are some conflicting results regarding the timing of ICSI. It has been reported that a preincubation period between oocyte retrieval and injection in ICSI cycles improved the percentage of mature oocytes [5, 6], the fertilization rate [1, 6, 7], and the embryo quality [1, 2]. A long oocyte preincubation (9–11 hours) prior to ICSI is thought to have bad effects on embryo quality [2], probably due to oocyte ageing. However, other studies supported different results as no statistically significant differences in the fertilization [3, 4] or the pregnancy rates [2, 6] were found in preincubated oocytes during ICSI cycles. 

In theory, some problems may be associated with the injection time. Oocytes are retrieved prior to ovulation in the procedure of IVF or ICSI. According to some reports [13, 14], preovulatory oocytes are not fully mature, even though a first polar body is present. It is so called the cytoplasmic immaturity. Cytoplasmic maturity is thought to be asynchronous with nuclear maturity in stimulated cycles [15, 16]. Hence, the fertilizing ability of an oocyte with a mature nucleus is not necessarily at its maximum potential. Therefore preincubation of oocytes prior to IVF or ICSI may induce cytoplasmic maturation that could eventually increase fertilization and also pregnancy rates. Moreover, Balakier and colleagues [14] reported that human oocytes progressively develop the ability for full activation and normal development during the MII arrest stage. The improvement in fertilization rates was obtained when ICSI was carried out 6–8 hours after the first polar body expulsion. For normal fertilization to occur, both nuclear and cytoplasmic maturity is required independently [15].

Whether to carry out the oocyte denudation directly after their retrieval or to keep the surrounding cumulus cells during the preincubation is also not clear in the literature. The only published report focusing on the influence of denudation timing did not show any significant influence on the results [3].

The purpose of this study was to analyze retrospectively the impact of oocyte denudation and microinjection timings on the ICSI outcome in a selected population so as to carry out corrective measures to improve the results.

## 2. Material and Methods

### 2.1. Patients

ICSI were performed at Cochin-Saint Vincent Hospital (Paris, France) between January 2004 and February 2007. Only were included in this retrospective study ICSI with the following parameters: attempt rank 1 or 2 of ICSI; female age < 36 years old; male factor infertility (total motile spermatozoa after selection <500 000 or presence of antispermatozoa antibodies IgG > 80% and/or IgA > 80% located on sperm head) long protocol using GnRH and rFSH for ovarian stimulation [17]; oocytes retrieval performed  36.5 ± 1  hour after hCG administration; use for ICSI of freshly ejaculated sperm.

We excluded from this retrospective study cycles where a female infertility could be evidenced as: (i) premature ovarian failure (FSH at Day 3 ≥ 10 IU/mL), (ii) <4 oocytes retrieved, (iii) grade III or IV endometriosis according to American Fertility Society classification, or (iv) polycystic ovaries syndrome (ESHRE 2003). We also excluded the cycles during which a long ICSI procedure was mentioned on the laboratory sheet (more than one hour) or ICSI for which more than 15 oocytes were collected to avoid including polycystic ovary or overstimulated patients who might have an underlying oocyte maturation problem. For these reasons and to eliminate a potential bias linked to the sperm origin, ICSI performed with surgically retrieved spermatozoa was excluded from the study.

### 2.2. Ovarian Stimulation

Ovarian stimulation, ovulation triggering, and oocytes collection were carried out as described elsewhere [17].

### 2.3. Oocyte Preparation

The cumulus and corona cells were removed using enzymatic digestion after a variable timing following oocyte retrieval then incubated in IVF medium (Medicult, France) at 37°C, 5% CO_2_ in air till ICSI was performed. Only morphologically normal-appearing mature oocytes with a visible first polar body by the time of ICSI procedure were microinjected. During all the time of the current study, the ICSI conditions were identical (equipments, media, and culture conditions).

### 2.4. Semen Preparation

Semen samples were collected the day of oocyte retrieval by masturbation. After liquefaction, semen was prepared by a 90-45 gradient system using Puresperm (JCD, France). Sperm motility and concentration were assessed according to World Health Organization [18] criteria before and after preparation. The timing between the end of sperm preparation and the beginning of ICSI was comprised between 30 minutes and 2 hours.

### 2.5. ICSI Procedure

Intracytoplasmic sperm injection was performed as described elsewhere [19]. Sperm injections were performed throughout the day, depending of the number of ICSI procedures, and the workload in the laboratory.

### 2.6. Timing

**Figure 1:**
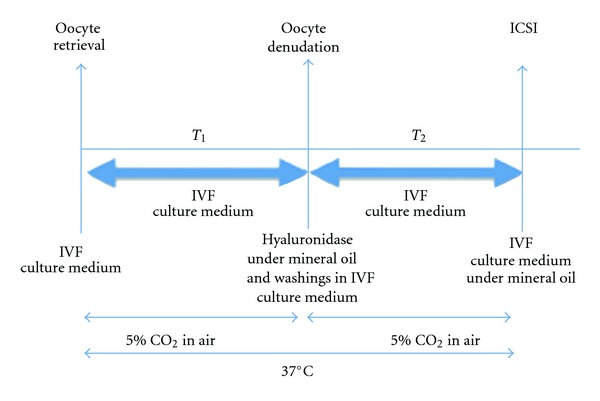
Schematic representation of the two timings *T*_1_ and *T*_2_.  *T*_1_: Time elapsed between oocyte retrieval and denudation. *T*_2_: Time elapsed between oocyte denudation and ICSI.

Different timings were analyzed in this retrospective study (Figure 1). The timing between oocyte retrieval and oocytes denudation was the timing elapsed between the beginning of oocyte retrieval and the beginning of oocyte denudation (*T*_1_). The timing between oocyte denudation and ICSI procedure was the timing elapsed between the beginning of oocyte denudation and of ICSI procedures (*T*_2_). The timing was recorded immediately before each act. The timing of case assignment was random, determined only by the work load on a given day.

### 2.7. Assessment of Fertilization and Embryo Development

Assessment of fertilization was made 17-18 h after ICSI by checking the number of polar bodies and pronuclei. The fertilization rate was defined as the ratio between the number of diploid zygotes and the number of mature oocytes. After confirmation of fertilization, each normally fertilized oocyte was transferred into a new ISM1 medium 30 *μ*L droplet. Early cleavage was assessed 25 ± 1 hours after ICSI and embryo cleavage was evaluated after a 44 ± 2 hours culture. The cleavage rate was defined as the ratio between the number of embryos and the number of mature oocytes. Embryo grading was performed according to the number and the size of the blastomeres (regular or irregular cleavage), the presence or not of multinuclear blastomeres as well as the percentage of anucleate fragments. Embryos were put into one of 4 categories according to the percentage of anucleate fragments: type A, when there was no anucleate fragmentation, type B when 1–20% of the embryo was fragmented, C when the proportion of fragmentation ranged between 21 and 50% and D when over 50%, of the embryo was fragmented. Embryos were transferred 2 days after oocyte collection.

We considered as “TOP” embryos those seen fertilized at day 1 and were regular 4 to 5 cell embryos at day 2 with less than 20% fragmentation and without any multinuclear blastomeres. The percentage of TOP embryos was defined as the ratio between the number of top embryos and the total number of embryos.

There were different operators who participated to the gamete and embryo manipulations during the study. There was no difference in the fertilization and implantation rates between the different operators. 

### 2.8. Clinical Pregnancy and Implantation

Pregnancy test using serum hCG assay was performed on Day 12 after embryo transfer. Ongoing pregnancy refers to successful progress of pregnancy beyond the 10–12th week of gestation by an ultrasonography confirmation of one or more gestational sacs with heart activity.

The implantation rate (IR) was the ratio between the number of gestational sacs and the number of transferred embryos.

### 2.9. Statistical Analysis

Quantitative variables are reported as mean-plus or-minus standard deviation, while qualitative variables are reported as frequencies and percentages.

To study the impact of the two timings on various outcomes (fertilization rate, cleavage rate, and embryo quality), logistic regression models were used. Because each patient in our dataset could provide more than one datum, independence of the observations was questionable, and generalized estimated equations were used with exchangeable correlation matrices.

For each outcome, in a first step, we successively constructed (i) a logistic regression model to study the impact of the timing between oocytes retrieval and denudation and (ii) another model to study the impact of timing between oocytes denudation and ICSI injection. To account for potential nonlinear effects of both timings, second and third order polynomial terms were entered together with linear terms. Effects were considered significant if the corresponding *P* values were less than 0.05. Then, all significant terms obtained in the first step along with their interactions were included in another logistic regression model to study the independent impact of both timings. Again, effects were considered significant if the corresponding *P* values were less than 0.05.

## 3. Results

### 3.1. Patients and ICSI Cycles

A total of 110 of the 1691 (6.5%) ICSI cycles performed during this period met the criteria of inclusion. The results of ICSI from 110 treatment cycles are shown on Table 1. Mean sperm parameters after selection were as follows: 10.1.10^6^ ± 1.4 sperm concentration, 40.6 ± 2.9% progressive motility. They were not significantly different between each timing investigated (data not shown). A total of 1230 oocytes were obtained (11 ± 3.0 oocytes per retrieval), 903 oocytes (73.4%) were mature just before ICSI. The overall fertilization rate (FR) of the injected oocytes was 65.8% and 593 embryos (65.7% of cleavage rate) were obtained, with 25.8% of them considered as TOP embryos. Forty-six of the injected oocytes were not mature at denudation and so matured *in vitro* between denudation and ICSI. They represented 5.0% of the injected oocytes and their proportion did not significantly varied according to the different timings. The FR of the *in vitro* maturated oocytes (22/46 47.8%) was significantly lower comparing to the ones already mature at denudation (572/857 66.7%; *P* = 0.008, *χ* ^2^ test).

A total of 107 embryo transfers were performed with a mean number of 1.7 ± 0.3 embryos transferred (196 transferred embryos). These transfers led to 56 fetal sacs, 47 ongoing pregnancies, 37 live births, 7 miscarriages, 1 neonatal death following eclampsia, 1 unknown outcome, and 1 medical abortion for oligohydramnion.

### 3.2. Effect on the ICSI Parameters

**Figure 2:**
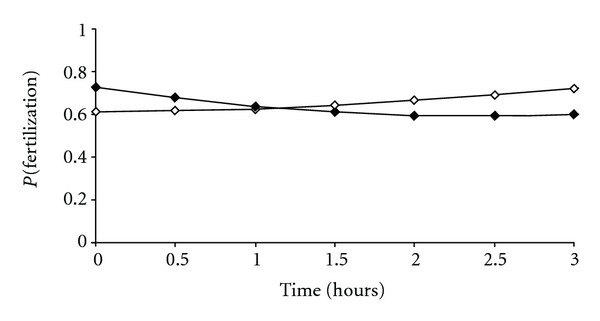
Influence of the timing between oocyte retrieval and denudation (white box) and the timing between oocyte denudation and ICSI injection (black box) on the fertilization rate. The relation between *P*(fertilization) (*P*(*f*), the probability of the oocytes to be fertilized) and the time between oocyte retrieval and denudation (*T*_1_) was statistically significant with a *P* value <0.0001 related to (*T*_1_)^2^. log⁡⁡(*P*(*f*))/(1 − *P*(*f*)) = 0.46 + 0.06 (*T*_1_)^2^. The relation between *P*(fertilization) (*P*(*f*), the probability of the oocytes to be fertilized) and the time between denudation and microinjection (*T*_2_) was statistically significant and linear with a 0.0083 *P* value related to *T*_2_, following the equation log⁡(*P*(*f*))/(1 − *P*(*f*)) = 0.97 − 0.50(*T*_2_) + 0.11 (*T*_2_)^2^and with a 0.005 *P* value related to (*T*_2_)^2^.

The statistical analysis of the data showed no statistical differences according to the time of denudation and microinjection on the MII percentage (data not shown). The fertilization probability *P*(*f*) significantly increased with the increase of *T*_1_ (*P* < 0.0001; Figure 2). The time *T*_1_ had consequently a significant effect on the fertilization but this effect was not linear. The maximum of probability of fertilization was at an interval of 3 hours between oocytes retrieval and denudation. On the other hand, the relation binding the time between oocyte denudation and microinjection (*T*_2_), *P*(*f*) was linear and statistically significant (Figure 2). But on the contrary the fertilization probability decreased with the increase of the time lasting between oocyte denudation and injection so the shorter *T*_2_ was, the better fertilization results that we had. Consequently, the combined effects of both periods *T*_1_ and *T*_2_ were influencing significantly the fertilization outcome. So, according to the fertilization rate results, the optimal timings are to perform the oocyte denudation within 3 hours after oocyte retrieval and to perform sperm microinjection without any delay after oocyte denudation.

In terms of embryo quality, there was not any statistical significant influence of both procedure timings on the percentages of TOP quality embryos (data not shown).

### 3.3. Effect on the ICSI Outcomes

**Figure 3:**
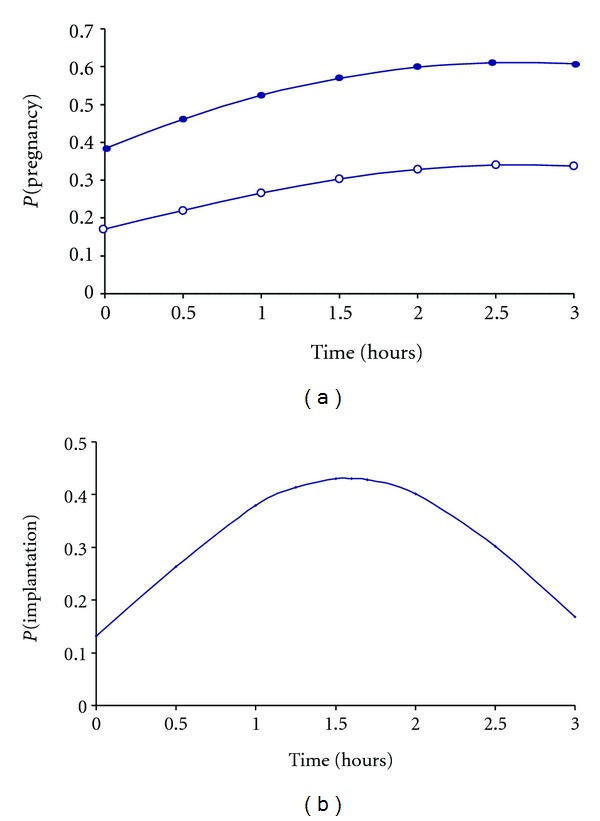
Influence of the timing between oocyte retrieval and denudation on the pregnancy rate (a) and the implantation rate (b). (a) The relation between the probability of pregnancy *P*(Pregn) and the time between oocyte retrieval and denudation (*T*_1_) follows the equation log⁡(*P*(Pregn))/(1 − *P*(Pregn)) = −2.69 + 0.70(*T*_1_) − 0.13(*T*_1_)^2^ + 1.11 but was not statistically significant (*P* values: (*T*_1_): 0.1364, (*T*_1_)^2^: 0.1232). Two curves are represented depending on how many embryos are transferred *R* = 1 (white circle) if 1 embryo is transferred and R = 2 (black circle) if 2 embryos are transferred. (b) The relation between the probability of implantation P(i) and the time between oocyte retrieval and denudation (*T*_1_) was statistically significant following the equation log⁡(*P*(i))/(1 − *P*(i)) = −1.89 + 2.04(*T*_1_) − 0.65(*T*_1_)^2^ (*P* values: (*T*_1_): 0.03 and (*T*_1_)^2^ 0.03). The best implantation rate was observed for a time between oocyte retrieval and denudation around 1.5 hours.

In terms of pregnancy results, both *T*_1_ and *T*_2_ did not have any statistically significant influence on pregnancy outcomes (data not shown for *T*_2_) but when representing the probability of pregnancy according to the time elapsing between oocyte retrieval and denudation (Figure 3(a)), it appeared that we had optimal results when denudation is achieved around 2 hours after oocyte retrieval. When considering the probability of delivery according to the same timings, it followed the same tendency. The same effect was observed whether one or two embryos were transferred with a higher pregnancy rates reached at 2 hours.

The time elapsed between oocyte retrieval and denudation had a significant impact on implantation rates with optimal results when oocytes were denudated around 1.5–2 hours after retrieval (Figure 3(b)). With regards to the time elapsed between denudation and microinjection, there was no statistically significant impact on the implantation results (data not shown).

## 4. Discussion

In the literature, among six studies interested in ICSI procedure timings [1, 2, 4–7], none analyzed the impact of both oocyte denudation and microinjection timings on ICSI outcomes. Most of them assumed that oocytes should be incubated *in vitro* surrounded by the corona and cumulus cells [1, 2, 6, 7]. Moreover, they stated that the denudation was achieved directly prior to ICSI but there is no evidence showing whether it is preferable to keep the cells surrounding the oocytes during preincubation and which timing it should be adopted. The originality of our study design was to separate the timing between the procedures into two successive periods: *T*_1_ and *T*_2_, to study distinctly the optimal timing of each procedure to improve ICSI results. Accounting for all the results, the denudation should be achieved at least 2 hours and up to 3 hours after oocyte retrieval for optimal fertilization and implantation results. ICSI should be achieved as soon as the denudation is completed.

We confirmed previous report [3], as we did not find any influence of the time between oocyte retrieval and denudation nor between denudation and ICSI on the percentage of meiotically mature oocytes. Other studies found that the preincubation of oocytes *in vitro* prior to denudation and/or injection is beneficial as we get more mature (MII) oocytes [5, 6]. Ho et al. [5] even stated that it was the only improvement that he got from pre-incubating oocytes prior to ICSI.

Even if no differences were observed in the percentage of meiotically mature oocytes in our study, significant differences were observed in fertilization rates. A longer incubation period prior to denudation (up to 3 hours) gave better fertilization rates. This could mean that a delay of denudation may not necessarily increase the number of meiotically mature eggs but it allows the MII oocytes to complete cytoplasmic maturation. Nuclear maturity can easily be assessed before ICSI as it is evidenced by the expulsion of the first polar body but the cytoplasmic maturity process is not very well known, it is thought to involve maternal mRNA and proteins [4]. In natural cycles, nuclear and cytoplasmic maturity is highly coordinated whereas in stimulated cycles, the two phenomena appear to be asynchronous [15, 16]. Hence, some oocytes retrieved from stimulated cycles could be cytoplasmically immature despite reaching the MII stage. This could be one explanation for the fact that the timing between oocyte retrieval and denudation influences significantly the implantation rate (with best results when denudation is achieved around 1.5–2 hours after oocyte retrieval) even though the embryo quality was not affected by the timing of the different procedures. The surrounding cells might also secrete paracrine substances and growth factors or express adhesion molecules on their surface membranes that might play a role in the nuclear and/or oocyte maturation. For example, the ovarian brain-derived neurotrophic factor secreted by granulosa and cumulus cells is essential for nuclear and cytoplasmic oocyte development [20].

Conflicting results concerning the preincubation of oocytes prior to ICSI were found in the literature. Some supported it [1, 2, 5, 6], others did not find evidence showing any advantages [3, 4]. And, even among those studies which support the incubation of oocytes prior to ICSI, it does not always concern the same parameters, upregulating either the fertilization rate only [1, 7], the embryo quality only [2], or both fertilization and pregnancy rates [6]; (our study). Furthermore, there is no common agreement on how many hours the oocytes should be incubated. We intentionally analyzed data of ICSI performed on a selected population to avoid possible factors that could bias the results. Among them, a fixed timing after hCG administration was determined (36.5 h ±  1 post hCG). In fact, the majority of the oocyte retrievals were performed between 36.0 and 37.0 hours, that is, 36.5 h ±  0.5 post HCG as only 13 (11.8%) was out of range. But the same conclusion was obtained after reanalysing the data without these 13 attempts. The time between oocyte retrieval and ICSI should not also exceed a certain sensible limit. In some studies, oocytes were injected up to 11 hours [1, 2] after retrieval, which seemed to have a bad influence on the embryo quality as significantly less good quality embryos were obtained from the oocytes injected 9–11 hours after retrieval [2]. It has been shown that ageing oocytes are much more sensitive to parthenogenetic activation [21] or that a longer preincubation period could affect oocyte quality [22]. The capacity of the cytoplasm of MII oocytes to decondensate sperm DNA, resume meiosis, and promote the evolution of male pronucleus appears to decline progressively 24 hours after oocyte retrieval [23]. Van de Velde claimed that ICSI should not be done more than 12 hours after retrieval because *in vitro* ageing seems to result in spindle instability and subsequent loss or scattering of chromosomes in the oocyte [24–26].

## 5. Conclusion

Our results suggest that the preincubation between oocyte collection and denudation up to 3 hours after retrieval in ICSI may not increase the percentage of mature oocytes but improves the fertilization and implantation rates even though the embryo quality is not influenced. On the other hand, the sperm injection should be achieved without any delay after oocyte denudation to keep good fertilization results.

## Figures and Tables

**Table 1:** ICSI cycle characteristics and outcomes.

Number of cycles	110
Number of oocytes (mean [range])	1230 (11.2 [5–27])
Number of MII oocytes at the injection (%) (mean [range])	903 (73.4%) (8.2 [4–15])
2PN fertilized oocytes (%) (mean [range])	594 (65.8%) (5.4 [4–15])
Number of degenerated MII after the injection (%) (mean [range])	43 (4.8%) (0.4 [0–4])
Number of obtained embryos (%) (mean [range])	593 (65.7%) (5.4 [0–14])
Number of TOP embryos (%) (mean [range])	153 (25.8%) (1.4 [0–7])
Number of transferred embryos (mean [range])	196 (1.8 [0–2])
Number of gestational sacs	56
Implantation rate	28.5%
Number of clinical pregnancies (per cycle %)	47 (42.7%)
Clinical pregnancy per embryo transfer	43.9%
Live birth (per cycle %)	37 (34.6%)

## References

[1] Rienzi L, Ubaldi F, Anniballo R, Cerulo G, Greco E (1998). Preincubation of human oocytes may improve fertilization and embryo quality after intracytoplasmic sperm injection. *Human Reproduction*.

[2] Yanagida K, Yazawa H, Katayose H, Suzuki K, Hoshi K, Sato A (1998). Influence of oocyte preincubation time on fertilization after intracytoplasmic sperm injection. *Human Reproduction*.

[3] van de Velde H, de Vos A, Joris H, Nagy ZR, van Steirteghem AC (1998). Effect of timing of oocyte denudation and micro-injection on survival, fertilization and embryo quality after intracytoplasmic sperm injection. *Human Reproduction*.

[4] Jacobs M, Stolwijk AM, Wetzels AM (2001). The effect of insemination/injection time on the results of IVF and ICSI. *Human Reproduction*.

[5] Ho JY, Chen MJ, Yi YC, Guu HF, Ho ES (2003). The effect of preincubation period of oocytes on nuclear maturity, fertilization rate, embryo quality, and pregnancy outcome in IVF and ICSI 1. *Journal of Assisted Reproduction and Genetics*.

[6] Isiklar A, Mercan R, Balaban B, Alatas C, Aksoy S, Urman B (2004). Impact of oocyte pre-incubation time on fertilization, embryo quality and pregnancy rate after intracytoplasmic sperm injection. *Reproductive BioMedicine Online*.

[7] Dozortsev D, Nagy P, Abdelmassih S (2004). The optimal time for intracytoplasmic sperm injection in the human is from 37 to 41 hours after administration of human chorionic gonadotropin. *Fertility and Sterility*.

[8] Falcone P, Gambera L, Pisoni M (2008). Correlation between oocyte preincubation time and pregnancy rate after intracytoplasmic sperm injection. *Gynecological Endocrinology*.

[9] Trounson AO, Mohr LR, Wood C, Leeton JF (1982). Effect of delayed insemination on in-vitro fertilization, culture and transfer of human embryos. *Journal of Reproduction and Fertility*.

[10] Harrison KL, Wilson LM, Breen TM, Pope AK, Cummins JM, Hennessey JF (1988). Fertilization of human oocytes in relation to varying delay before insemination. *Fertility and Sterility*.

[11] Veeck LL (1988). Oocyte assessment and biological performance. *Annals of the New York Academy of Sciences*.

[12] Khan I, Staessen C, van Den Abbeel E (1989). Time of insemination and its effect on in-vitro fertilization, cleavage and pregnancy rates in GnRH agonist/HMG-stimulated cycles. *Human Reproduction*.

[13] Kubiak JZ (1989). Mouse oocytes gradually develop the capacity for activation during the metaphase II arrest. *Developmental Biology*.

[14] Balakier H, Sojecki A, Motamedi G, Librach C (2004). Time-dependent capability of human oocytes for activation and pronuclear formation during metaphase II arrest. *Human Reproduction*.

[15] Eppig JJ, Schultz RM, O’Brien M, Chesnel F (1994). Relationship between the developmental programs controlling nuclear and cytoplasmic maturation of mouse oocytes. *Developmental Biology*.

[16] Sundstrom P, Nilsson BO (1988). Meiotic and cytoplasmic maturation of oocytes collected in stimulated cycles is asynchronous. *Human Reproduction*.

[17] Papageorgiou T, Guibert J, Goffinet F (2002). Percentile curves of serum estradiol levels during controlled ovarian stimulation in 905 cycles stimulated with recombinant FSH show that high estradiol is not detrimental to IVF outcome. *Human Reproduction*.

[18] (2001). [Laboratory manual of the WHO for the examination of human semen and sperm-cervical mucus interaction]. *Annali dell'Istituto Superiore di Sanita*.

[19] Fauque P, Guibert J, Jouannet P, Patrat C (2008). Successful delivery after the transfer of embryos obtained from a cohort of incompletely in vivo matured oocytes at retrieval time. *Fertility and Sterility*.

[20] Kawamura K, Kawamura N, Mulders SM, Gelpke MDS, Hsueh AJ (2005). Ovarian brain-derived neurotrophic factor (BDNF) promotes the development of oocytes into preimplantation embryos. *Proceedings of the National Academy of Sciences of the United States of America*.

[21] Plachot M, Crozet N (1992). Fertilization abnormalities in human in-vitro fertilization. *Human Reproduction*.

[22] Tesarik J, Sousa M, Testart J (1994). Human oocyte activation after intracytoplasmic sperm injection. *Human Reproduction*.

[23] Nagy ZP, Joris H, Liu J, Staessen C, Devroey P, van Steirteghem AC (1993). Intracytoplasmic single sperm injection of 1-day-old unfertilized human oocytes. *Human Reproduction*.

[24] Pickering SJ, Johnson MH, Braude PR, Houliston E (1988). Cytoskeletal organization in fresh, aged and spontaneously activated human oocytes. *Human Reproduction*.

[25] van Wissen B, Bomsel-Helmreich O, Debey P, Eisenberg C, Pennehouat G (1991). Fertilization and ageing processes in non-divided human oocytes after GnRHa treatment: an analysis of individual oocytes. *Human Reproduction*.

[26] Martini E, Flaherty SP, Swann NJ, Payne D, Matthews CD (1997). Analysis of unfertilized oocytes subjected to intracytoplasmic sperm injection using two rounds of fluorescence in-situ hybridization and probes to five chromosomes. *Human Reproduction*.

